# Evaluation of Cytotoxic and Antimicrobial Properties of Iranian Sea Salts: An *In Vitro* Study

**DOI:** 10.1155/2021/8495596

**Published:** 2021-12-14

**Authors:** Mohammad Nima Motallaei, Mohsen Yazdanian, Hamid Tebyaniyan, Elahe Tahmasebi, Mostafa Alam, Kamyar Abbasi, Reza Ranjbar, Alireza Yazdanian, Mehrdad Moosazadeh Moghaddam, Hamid Sedighian

**Affiliations:** ^1^Research Center for Prevention of Oral and Dental Diseases, Baqiyatallah University of Medical Sciences, Tehran, Iran; ^2^Islamic Azad University, Science and Research Branch, Tehran, Iran; ^3^Department of Oral and Maxillofacial Surgery, School of Dentistry, Shahid Beheshti University of Medical Sciences, Tehran, Iran; ^4^Department of Prosthodontics, School of Dentistry, Shahid Beheshti University of Medical Sciences, Tehran, Iran; ^5^School of Dentistry, Baqiyatallah University of Medical Sciences, Tehran, Iran; ^6^Department of Veterinary, Science and Research Branch, Islamic Azad University, Tehran, Iran; ^7^Applied Biotechnology Research Center, Baqiyatallah University of Medical Sciences, Tehran, Iran; ^8^Applied Microbiology Research Center, Baqiyatallah University of Medical Sciences, Tehran, Iran

## Abstract

**Background:**

Dental caries is known as a multimicrobial disease. Caries are very prevalent in numerous countries, and the incidence is higher in underdeveloped countries than in developed countries. Dental caries is a major public health problem, and it is the most prevalent health problem across the world, affecting 2.4 billion people. Natural mouthwashes can be beneficial in the prevention of dental caries and oral infections without the side effects of synthetic mouthwashes.

**Aim:**

The aim of the present study was to investigate the antibacterial, antifungal, and cytotoxicity properties of sea salt from different areas of Iran.

**Methods and Materials:**

Sea salts from different areas (Urmia, Qom, and Jarquyeh) of Iran were collected. In order to define the elemental and mineralogical features of different salt samples, X-ray powder diffraction (XRD) was employed. Different concentrations (0.19–50 mg/mL) of sea salt were used in the antimicrobial and antibiofilm tests. The antimicrobial (MIC, MBC, MFC, and DAD tests) and antibiofilm (formation and degradation tests) effects were evaluated against *L. acidophilus, S. aureus, E. coli, S. mitis, S. mutans, S. salivarius*, and *C. albicans.* The cytotoxic effect of salts was evaluated on human gingival fibroblasts by the MTT test.

**Results:**

The range of MIC values in mg ml^−1^ was as follows: *S. salivarius* (50), *S. mutans* (50), *S. mitis* (50), L. *acidophilus* (12.5 to >50), *C. albicans* (50), *E. coli* (12.5 to 25), and *S. aureus* (12.5 to 25), while MBC values were, *S. mutans* (>50), S. salivarius (>50), *S. mitis* (>50), *L. acidophilus* (50 to >50), *C. albicans* (>50), *E. coli* (50), and *S. aureus* (50). MTT results showed that more than 50% of cell viability depends on decreasing the salt concentration (<1.56 mg/ml).

**Conclusion:**

Sea salts had significant antimicrobial effects on cariogenic bacteria and *C. albicans*. Therefore, sea salts can be a suitable candidate for mouthwash.

## 1. Introduction

Dental caries is a multifactorial disease which is considered by local destruction of the tooth. Dental biofilm plays an important role in the progression of periodontal diseases and caries [1]. Evidence from the decayed, missing, and filled index (DMF), published by FDI (Federation Dental International), presented that carious lesions are highly prevalent in many countries and are higher in underdeveloped countries than in developed countries. Therefore, dental caries is a major public health problem [2]. A microbial biofilm contains a microbiological community. They are organized into an extracellular matrix. They stick to tooth surfaces. Poor oral hygiene and high consumption of fermentable carbohydrates cause an imbalance in the biofilm. These situations cause demineralization [3]. An important factor to initiate carious is the presence of bacteria, mostly *Streptococcus mutans*, though it is not sufficient for caries development. In carious lesions, additional microbial species were also isolated such as Gram-negative bacteria *lactobacilli*. They have been related to the dental caries [1, 4–6]. The metabolism of these species of bacteria produces acids, which decrease pH and result in demineralization of the tooth tissue [6]. *Streptococcus mutans*'s capability to make extracellular polysaccharides (mainly glucans) is an essential factor for tooth caries [1, 3, 5]. The production of acids, especially lactic acid, is a very significant virulence factor of *S. mutans* which leads to caries and an acidic environment [5, 7]. The acidic environment determined by the bacterial plaque decalcifies the enamel and/or dentine [6, 7]. *Lactobacilli* should be considered as “secondary invaders.” They are not capable of producing caries [3, 5–7]. Bacteria produce glucans from nutritional carbohydrates by glucosyltransferases (GTFs). Glucans are important for oral pathogens to stick to and accumulate on the tooth surface [1]. Many efforts have been made to remove cariogenic microorganisms from the oral cavity. In preventing dental caries antimicrobials such as ampicillin, penicillin, and tetracycline have been very effective. However, prolonged application of these substances causes unwanted side effects such as microorganisms' susceptibility, diarrhea, vomiting, and tooth staining. Sanguinarine harvest from the *Sanguinaria canadensis*. It has an extensive range of action against several oral bacteria. It has been used in different oral care products due to its robust antibacterial usefulness. It was reported to be related with oral leukoplakia, therefore its application had been decreased. These complications necessitate more research for natural antibacterial materials which are specific for oral microorganisms and safe for humans [8, 9]. Chemical agents have potential side effects. For example, chlorhexidine gluconate (0.12%) could result in dryness, staining of teeth, loss of taste sensation, and allergies. Therefore, there is a need for tissue friendly and cost-effective rinses. Saltwater rinses have been used after extractions, alveolar osteitis, and oral infections [10]. Saltwater rinses inducing vasodilation and lowering the bacterial load, simplifying phagocytes to the injury site, alkalizing saliva, and acting as an astringent [10]. Furthermore, saltwater improves wound healing through reducing inflammation and contracting the tissues [11]. There is very little available data on the efficiency of sea salt. Sea salt is produced via evaporation of saltwater lakes or oceans with very little processing, but table salt is typically gained from underground salt deposits and severely processed [10, 12, 13]. Refined salt typically contains about 99.5–99.9% NaCl and some additives such as anticaking agents and whitening. “Natural” sea salt contains no additives and is not chemically processed. This salt is naturally evaporated by the sun allowing the sea salt to hold its natural mineral content and some trace heavy metals [10, 12, 13]. The aim of this study was to evaluate the efficacy of sea salts from different origins in Iran in controlling some oral microorganisms and their effect on normal human gingival fibroblast cells.

## 2. Materials and Methods

### 2.1. Materials

*Streptococcus salivarius*, *Streptococcus mutans*, *Streptococcus mitis*, *Candida albicans*, *Lactobacillus acidophilus*, *Staphylococcus aureus*, *and* Escherichia coli, and human gingival fibroblast cells were gifted. Brain heart infusion (BHI) broth and agar and yeast peptone dextrose (YPD) broth and agar and crystal violet were provided from Merck (Darmstadt, Germany). The MTT Kit was purchased from Bioidea (Iran). Dimethyl sulfoxide (DMSO) was purchased from Sigma‐Aldrich. Dulbecco's modified Eagle's medium (DMEM), trypsin, fetal bovine serum (FBS), phosphate‐buffered saline (PBS), antistreptomycin, and betaglycerol were bought from Gibco (New York, USA).

### 2.2. Sea Salt Sampling

#### 2.2.1. Sea Salt Sampling

**Figure 1:**
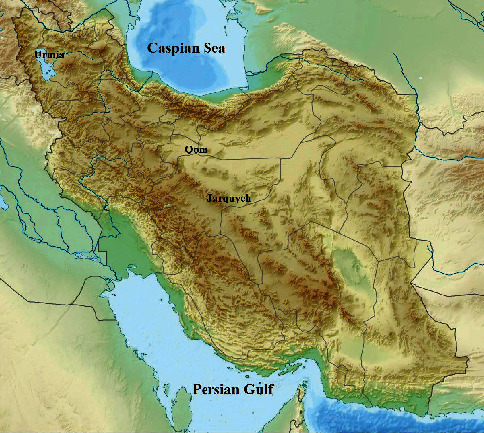
Three samples were collected from different regions of Iran.

Sea salts were collected in 2020 from Urmia (West Azerbaijan) (37.6822° N, 45.3943° E), Qom (34.5239° N, 51.8946° E), and Jarquyeh (Isfahan Province) (32.09548° N, 52.74346° E) (Figure 1).

#### 2.2.2. Identification of Sea Salt Mineral Elements

Elemental characteristics of salt samples were characterized by X-ray powder diffraction (XRD) (using a Philips PW3710 X-ray equipped with Cu K*α* radiation and a secondary graphite monochromator at 40 kV and a current of 30 mA in a range from 2 to 70° 2*θ*/degree, with a speed of 3.0 2 *θ*/min). Patterns were recognized by X'Pert HighScore Plus with a PAN-ICSD database [12].

### 2.3. Cytotoxic Evaluation

Human gingival fibroblasts were incubated in 96-well plates with different concentrations (0.19–50 mg/mL) of sea salt in the growth medium. Cell survival was analyzed in this test containing 3-(4,5-dimethylthiazol-2-yl) 2,5-diphenyl tetrazolium bromide. 2 × 10^5^ cell/ml of the fibroblast cells were plated in each well. Then, different concentrations of sea salt samples (0.19–50 mg ml^−1^) with DMEM (serum-free) were added (100 µl/well). Then, the cultures were incubated (37°C) (moistened atmosphere of 5% CO_2_) for 24 h. In the next step, using the MTT solution (5 mg/ml), cell growth induction activity was assessed. MTT solutions were added to each well, and the plates were incubated (4 h at 37°C) (moistened atmosphere of 5% CO_2_). Dimethylsulfoxide (DMSO) (1000 µl) was used to change the well's medium. DMSO dissolves the dark blue crystals. The plates were read with an ELISA reader (EL *X* 808) after 10 min (room temperature) at 570 nm as the test wavelength and 630 nm as the reference wavelength. An MTT-based method was used to analyse cell mitochondrial activity after 24 h and 48 h of conditioning. Data were changed into percentages of viable cells [14, 15]. The MTT assay measures the mitochondrial and metabolic activity of treated cells. The MTT assay is broadly known as a reliable method to study cell viability [14, 15]. The results were presented as percentages (control value = 100%). All tests were done three times. The percentages of cell viability were measured using the following equation:

(1) $$\begin{array}{c} \text{the percentage of cell viability}=\frac{\text{samples }\left(\text{OD}\right)}{\text{control }\left(\text{OD}\right)\;}\times 100. \end{array}$$

### 2.4. Antimicrobial Activity of the Sea Salts

#### 2.4.1. Bacterial Strain and Inoculum Preparation for Evaluation of Minimum Inhibitory (MIC), Minimum Bactericidal Concentration (MBC), and Minimum Fungicidal Concentration (MFC)

The bacterial and fungal strains used in this experiment were *Streptococcus mitis, Streptococcus mutans, Streptococcus salivarius, Lactobacillus acidophilus, Staphylococcus aureus*, *Escherichia coli*, and *Candida albicans.* The bacterial strains were reactivated in brain heart infusion agar (BHI) medium ((37°C, (5% CO_2_) (48 h)). Then, a loopful of bacteria were suspended in BHI Broth medium (25 mL) (Merck, Darmstadt, Germany). After incubation, the concentration of cells was determined (37°C for 24 h). In a spectrophotometer (at 625 nm) (absorbance of 0.18), a cell density equivalent of 1.0 × 10^8^ CFU/mL was obtained [16]. A concentration of 1.0 × 10^5^ CFU/mL was used for the MIC assay [4]. The stock culture of *C. albicans* was inoculated initially into sterilized yeast peptone dextrose (YPD) broth to form the *C. albicans* suspension. A concentration of 1.0 × 10^5^ CFU/mL was used for the MIC assay (18, 19).

#### 2.4.2. Determination of MIC, MBC, and MFC Tests

BHI and YPD broth (100 *μ*L) was injected into the wells of 96-well microtiter plates to assess MIC. In the next stage, sea salts (100 *μ*L) were inserted into the first column of the wells at their primary concentration (50 mg/ml). Then, by transferring well content (100 *μ*L) from the highest concentrated to the least concentrated, the sea salt solution was sequentially diluted (from 50 to 0.19 mg/mL) (1 : 1 v/v) [16]. In the next stage, 100 *μ*L of the well contents of the last column were thrown away. At the final stage, bacterial and fungal inoculums were inserted (100 *μ*L) (1.0 × 10^5^ CFU/mL). The control groups were used as follows: (1) growth control (only microbial content without any antimicrobials); (2) antimicrobial control (Chlorhexidine (CHX) 0.2%); and (3) sterility control (only sterile culture medium). The microplates were incubated (37°C, 5% CO_2_, and 24 h) [4].

#### 2.4.3. Disk Agar Diffusion Test (DAD)

The antimicrobial activity of sea salt solutions was determined by DAD using brain-heart infusion. Colonies of different strains grown on BHI agar were suspended in NaCl solution (145 mM). Then, they were adjusted to the McFarland 0.5 scale by a spectrophotometer. Sea salt solutions (400 ml) were mixed with BHI agar (40 ml) (at 45°C). Then, they poured on a set layer of BHI agar [17, 18]. Then, strains were inoculated on plates by sterile swabs [17, 18]. 0.5 ml of suspension of inoculums having 3 × 10^8^/ml of strains was streaked on BHI agar. By the DAD test, the antibacterial activity of sea salt solutions was measured. The plates of sea salt solutions were filled with 0.08 ml each of 2× MBC of each sea salt and 0.2% CHX (positive control). Then, plates were incubated (for 48 h) (at 37°C). The inhibition zone around the wells was measured and noted [17, 18].

#### 2.4.4. Biofilm Formation and Degradation Evaluation

Biofilm growth was assessed by the crystal violet staining method. The strains were cultured in microplates with sucrose (1%) and sterile BHI agar. Microplates were cultured with 2×MBC of each sea salt solution, under anaerobic conditions at 37°C with CO_2_ 5% for 48 h. Then, the broth was eliminated and the microplates were washed with PBS 3 times to remove nonadherent bacteria. Then, microplates were dried for 45 minutes at 60°C. Then, crystal violet (1% (w/v)) (100 *μ*L) solution was added to each well. Then, microplates were incubated for 15 minutes. Using PBS, the microplates were washed. By adding ethanol (95%) (125 *μ*L) to each well, biofilm formation was determined. With a microplate reader, the optical density of wells (OD) was measured in comparison to the control biofilm (without sea salt) at 590 nm [19]. The mean absorbances of the samples were assessed, and by the following formula, the percentage inhibition gained for the sea salt solutions at different concentrations was measured:

(2) $$\begin{array}{c} \text{the biofilm formation rate}=\frac{\text{samples }\left(\text{OD}\right)}{\text{control }\left(\text{OD}\right)}\times 100,\\ \text{the biofilm reduction rate}=100-\left(\frac{\text{samples }\left(\text{OD}\right)}{\text{control }\left(\text{OD}\right)}\right)\times 100, \end{array}$$

where OD _treatment_ and OD _control_ refer to the absorbance at 570 nm in each well with and without the samples, respectively, after the addition of the dissolving solution.

### 2.5. Statistical Analysis

The results were analyzed by Tukey post hoc test and one-way ANOVA to compare means among groups. Statistical evaluation becomes carried out with SPSS statistics model 20.

## 3. Results

### 3.1. Identification of Sea Salt Mineral Elements

The results for the mineral elements of the sea salt samples are found in Table 1.

### 3.2. Cell Viability Evaluation

**Figure 2:**
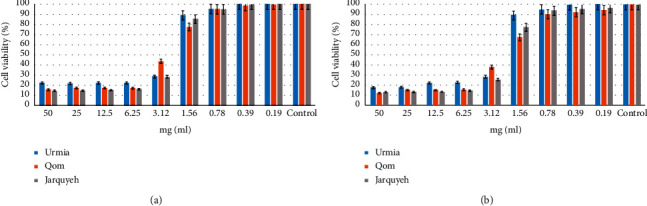
The percentage of cell viability by MTT exclusion on fibroblast cell lines. (a) 24 h and (b) 48 h. Data are expressed as mean ± SD (*n* = 3).

To determine the possible effect of sea salt samples on cell growth, cells were incubated with different extracts (0.19 to 50 mg/ml) (for 24 and 48 h). Using samples, a significant reduction of viable cells was observed in a dose and time-dependent pattern. By determination of the optical density of vital cells after treatment with the samples for incubation of 24 and 48 h, the cell viability percentage for both cell lines was calculated and compared to the percentage of the control group. The cell viability was increased by decreasing the concentration of salt, and the 1.56 mg/ml was considered as more than 50 percentage of cell viability. Results are shown in Figure 2.

### 3.3. Antimicrobial Analysis

#### 3.3.1. MIC

MIC values of the sea salt solutions against strains were determined by the broth microdilution method (Table 2). The range of MIC values in mg ml^−1^ was as follows: *S. salivarius* (50); *S. mutans* (50); *S. mitis* (50); L. *acidophilus* (12.5 to >50); *C. albicans* (50); *E. coli* (12.5 to 25); and *S. aureus* (12.5 to 25) (Table 2).

#### 3.3.2. MBC and MFC

The range of MFC and MBC values in mg mL^−1^ were *S. mutans* (>50); *S. salivarius* (>50); *S. mitis* (>50); *L. acidophilus* (50 and > 50); *C. albicans* (>50); *E. coli* (50); and *S. aureus* (50) (Table 2).

#### 3.3.3. Disk Agar Diffusion Analysis

The means of microbial growth inhibition zones. Results were depended on the samples and on the strains. The range of zones of microbial growth inhibition by sea salt samples was (mm) as follows: *S. mutans* (5 to 6 mm); *S. salivarius* (4 to 6 mm); *S. mitis* (4 to 6 mm); *L. acidophilus* (4 to 5 mm);*C. albicans* (4 to 5 mm); *E. coli* (3 to 4 mm); and *S. aureus* (4 to 5 mm) (Table 3).

#### 3.3.4. The Effect of Sea Salt Solutions on the Formation of Microbial Biofilm (Crystal Violet Staining Assay)

The effect of samples on the prevention of biofilm formation was tested using the microdilution method. Biofilm formation rates of samples are reported in percentages in Table 4. These percentages compare the biofilm formation of the tested microorganisms during exposure of different samples with the control group by measuring the OD of each well (at 570 nm wavelength) (Table 4). All salt solutions had antibiofilm properties. Sea salt solutions had relatively similar results.

#### 3.3.5. The Effect of Sea Salt Solutions on the Degradation of Microbial Biofilm (Crystal Violet Staining Assay)

The effect of treatment with samples on biofilms which had already been formed was tested using the same method. In this case, the biofilm reduction rate in percentage was calculated (Table 5). All salt solutions had antibiofilm properties. Sea salt solutions had relatively similar results.

## 4. Discussion

Some methods to control dental caries include the use of varnish, fluoride gel, professional tooth cleaning, antimicrobial agents, fissure sealant, fluoride mouthwashes, fluoride gels for personal use, fluoride supplements, oral hygiene, diet control, and noncariogenic sweeteners like xylitol. Natural products can also be used as coadjutant factors for caries control. Natural materials are a useful substitute to synthetic antimicrobials [16]. Many bacteria are involved in the carious procedure. The beginning of the caries is not dependent on the presence of *S. mutans* only. The *S. mutans*' biofilm is commonly selected to assess the antibacterial activities of various materials. Sucrose and *S. mutans* are the main factors of the growth of biofilms [5, 16]. Mechanical plaque control is the main method for plaque control, but it needs patient motivation and cooperation, so chemical agents can be useful adjutants for achieving plaque control. Chloride helps the host's defense against infection. It serves as a substrate for the production of chlorine bleach (microbicide) by stimulated neutrophils and helps maintain ionic homeostasis for antimicrobial activity inside phagosomes [11].

Sea salt makes the environment alkaline, thereby accelerating the healing of the surgical wound [7]. Sea salt increases the pH of the oral environment and helps reduce the acidity created by bacteria [7, 10, 11]. Also, saltwater decreases inflammation and contracts the tissues [11]. Saltwater induces vasodilation and helps phagocytes, lowering the bacterial load. Salt is recognized as osmosis kills some types of bacteria, effectively by sucking water out of them. Bacterial enzymes cannot function without water, and eventually, the cell can be collapsed [10].

In this study, the range of zones of microbial growth inhibition by sea salt samples was as follows: *S. mutans* (5 to 6 mm); *S. salivarius* (4 to 6 mm); *S. mitis* (4 to 6 mm); *L. acidophilus* (4 to 5 mm); *C. albicans* (4 to 5 mm); *E. coli* (3 to 4 mm); and *S. aureus* (4 to 5 mm). The best results were, respectively, related to Urmia, Qom, and Jarquyeh. In this study, we evaluate the effect of sea salts on the formation and degradation of microbial biofilm. Sample 2 (Qom) had the highest effect on the formation of biofilm, and sample 3 (Jarquyeh) had the lowest effect on the formation of biofilm. Sample 3 had the highest effect on the degradation of biofilm, and sample 2 had the lowest effect on the degradation of biofilm. MTT results showed that more than 50 percentage of cell viability depends on decreasing the salt concentration (≤1.56 mg/ml).

In this study, sea salts were collected from different regions in Iran. In this study, sea salts were effective against the tested microorganisms. Michel et al. assessed the usefulness of the application of sea salt rinse (each child rinsed with a solution containing 2.5 grams of sea salt in 20 ml of water) on periodontitis in street children of Manila [20]. Mani et al. studied 30 adults with gingivitis at a dental college in Maharashtra (India). There was a significant decrease in clinical indices in the group using sea salt rinse for three months compared to the other groups [13]. However, one study conducted by Hoover J et al. reported that application of sea salt rinse for 30 days was not useful for the treatment of periodontitis and gingivitis. This may be contributed to the fact that the study had a short trial period and a small sample size [10]. Further studies are needed to investigate the effects of sea salt. The limitations of this study are that it is an in vitro study and sea salt rinse needs to be verified clinically in future in vivo research studies. Although sea salt rinse has a significant effect on microbial organisms, it was less effective than chlorhexidine 0.2% mouthwash. Nevertheless, CHX mouthwash has many side effects, especially when used for a long time, causing taste changes, burning mouth, dry mouth, tooth staining, and side effects from probable swallowing. Using natural mouthwash without any significant side effects can be a helpful substitute, even with a lower biofilm impact and antimicrobial effect [21].

## 5. Conclusion

Antimicrobial studies specifically demonstrate that sea salt solutions are potent plaque inhibitors that could be used as a mouthwash. The solutions blocked plaque formation and degraded biofilm formation. However, further long-term clinical trials are required to include the much needed standardization and certification of the mouthwash in order to overcome the drawbacks of the gold standard chlorhexidine.

## Figures and Tables

**Table 1:** Sea salt mineral elements.

Elements (ppm)	S1	S2	S3
Ag	<0.1	<0.1	<0.1
Al	<0.1	<0.1	0.6165
As	2.292	3.404	1.8495
Be	<0.1	<0.1	<0.1
Ca	814.424	1615.198	2681.159
Cd	<0.1	<0.1	<0.1
Co	<0.1	<0.1	<0.1
Cr	<0.1	<0.1	<0.1
Cu	<0.1	<0.1	<0.1
Fe	<0.1	<0.1	<0.1
Hg	<0.1	<0.1	<0.1
K	640.232	349.761	1557.896
Mg	871.724	645.909	3685.437
Mn	<0.1	<0.1	<0.1
Ni	<0.1	<0.1	<0.1
P	6.876	7.659	5.5485
Pb	1.528	<0.1	<0.1
S	507.296	717.393	1131.278
F	<0.1	<0.1	<0.1
Sr	4.584	17.02	51.1695
V	0.764	<0.1	<0.1
Zn	<0.1	<0.1	<0.1

Urmia (S1), Qom (S2), and Jarquyeh (S3).

**Table 2:** MIC, MBC, and MFC in mg·mL^−1^ of sea salts obtained using the broth microdilution method.

Bacteria	Sample 1		Sample 2		Sample 3	
	MIC (mg/ml)	MBC/MFC (mg/ml)	MIC (mg/ml)	MBC/MFC (mg/ml)	MIC (mg/ml)	MBC/MFC (mg/ml)
*E. coli*	25	50	25	50	12.5	50
*S. aureus*	12.5	50	25	50	25	50
*S. mutans*	50	>50	50	>50	50	>50
*S. salivarius*	50	>50	50	>50	50	>50
*S. mitis*	50	>50	50	>50	50	>50
*L. acidophilus*	12.5	50	50	>50	25	50
*C. albicans*	50	>50	50	>50	50	>50

All samples were tested three times in independent experiments. Results show the insignificant difference between samples. Urmia (sample 1), Qom (sample 2), and Jarquyeh (sample 3).

**Table 3:** Mean area of microbial growth inhibition zones in mm (*n* = 3) provided by the sea salt samples.

Bacteria	MBC/MFC concentrations			CHX 0.2%
	Sample 1	Sample 2	Sample 3	
*E. coli*	4	3	4	15
*S. aureus*	4	4.5	5	17
*S. mutans*	6	5	5	18
*S. salivarius*	5.5	6	4	17
*S. mitis*	5	6	4	17
*L. acidophilus*	4	5	4	16
*C. albicans*	5	5	4	16

The difference was significant between the samples and control (CHX 0.2%) (*p* < 0.01). Results show the insignificant difference between samples. Urmia (sample 1), Qom (sample 2), and Jarquyeh (sample 3).

**Table 4:** The effect of sea salts on the biofilm formation of microbial biofilms (percentages).

Bacteria	OD (570 nm)			CHX 0.2 (%)
	Sample 1 (%)	Sample 2 (%)	Sample 3 (%)	
*E. coli*	85	90	85	20
*S. aureus*	85	90	85	22
*S. mutans*	90	85	80	20
*S. salivarius*	90	90	85	25
*S. mitis*	85	90	85	25
*L. acidophilus*	85	90	80	20
*C. albicans*	85	85	80	22

All samples were tested three times in independent experiments. The difference was significant between the samples and control (CHX 0.2%) (*p* < 0.01). Results show the insignificant difference between samples. Urmia (sample 1), Qom (sample 2), and Jarquyeh (sample 3).

**Table 5:** The effect of extracts on the degradation of microbial biofilms (percentages).

Bacteria	OD (570 nm)			CHX 0.2 (%)
	Sample 1 (%)	Sample 2 (%)	Sample 3 (%)	
*E. coli*	11	8	10	70
*S. aureus*	12	9	11	70
*S. mutans*	8	10	10	72
*S. salivarius*	10	10	11	75
*S. mitis*	11	10	11	73
*L. acidophilus*	12	9^*∗*^	13^*∗∗*^	71
*C. albicans*	7	10	11	70

All samples were tested three times in independent experiments. The difference was significant between the samples and control (CHX 0.2%) (*p* < 0.01). Results show the insignificant difference between samples. Urmia (sample 1), Qom (sample 2), and Jarquyeh (sample 3).

## Data Availability

All the data generated or analyzed during this study are included in this published article, and also the datasets analyzed to support the findings of this study are available from the corresponding author upon request.
